# Supplementary material for: What are data spaces? Systematic survey and future outlook
Source: Data Brief. 2024 Oct 1;57:110969. doi: 10.1016/j.dib.2024.110969 (PMC11827019; doi:10.1016/j.dib.2024.110969)
Supplement: Supplementary file 1 [file mmc1.pdf]

What are Data Spaces?
Systematic Survey and Future Outlook

Manlio Bacco 0000-0001-6733-1873

European Commission, Joint Research Centre (JRC), Ispra, Italy
Institute of Information Science and Technologies (ISTI), CNR, Pisa, Italy

Alexander Kocian 0000-0001-8847-0768
Stefano Chessa 0000-0002-1248-9478

Department of Computer Science, University of Pisa, Italy

Antonino Crivello 0000-0001-7238-2181
Paolo Barsocchi 0000-0002-6862-7593

Institute of Information Science and Technologies (ISTI), CNR, Pisa, Italy

Abstract

Data spaces, a novel concept pushing data sharing and exchange, are experiencing momentum because of recent developments motivated by the increasing need for interoperability and data sovereignty. After an initial phase, dating back to approximately twenty years ago, in which this concept has been tentatively explored in different scenarios, it is presently going through a consolidation phase in which both specifications and implementations converge towards a common reference for standardisation. In this context, we offer our view on data spaces by presenting a systematic literature survey, a description of the components needed to build them, how they work, and of existing mature software implementations. We thoroughly present the architectural vision behind the concept and we analyse the Reference Architectural Model by IDS. We provide practical pointers to readers interested in experimenting with software components used in data spaces, and we conclude by highlighting open challenges for their success.

*Corresponding author: Manlio Bacco, e-mail manlio.bacco@ec.europa.eu.
The opinions expressed are those of the author only and should not be considered representative of the European Commission's official position.

Keywords: data space, specifications, systematic survey, connectors, data management

1. Introduction

In 2005, the concept of data space was introduced in [1] as a response to the rapidly expanding demands of *data everywhere*. Data spaces represent a new abstraction for data management, to be implemented by means of Dataspace Support Platforms (DSSPs). A DSSP has the objective of facilitating data exchange and sharing in a wide variety of formats, of being accessible through a variety of different systems, and of allowing the use of different applications.

What was crucial, at that time, was defining the differences between data spaces, Data Bases (DBs), and other tools for data integration, whose evolution can be read in [2]. A comparison among the concepts we explore in this work is proposed in Table 1, and references can be found in what follows. Basically, a DB is a centralised collection of semantically homogeneous data in a rigid data management system [3], contrarily to a data space that is a collection of decentralised heterogeneous data. It must be noted, anyway, that DBs had already become increasingly flexible over time, as demonstrated by the advent of non-relational (e.g., NoSQL) database systems in the early 2000s. Since then, they have proved to be a solution for managing large and heterogeneous datasets, although with a lack of focus on data exchanges in federated settings. Non-relational DBs can be classified according to some characteristics [4], all sharing a limited (or none) use of schemas as instead common in relational DBs. In fact, they can be used every time data have a structure that does not fit well in the rigid structure of relational tables [5].

Going back to the need for a clearer definition of data spaces, we report its evolution in the following. In [1, 6, 7], a data space is described simply as a set of relationships and participants. In [8], it is defined as a large-scale heterogeneous collection of data distributed in several sources in various formats, with a mechanism to handle structured, semi-structured, and unstructured data. In [9], the focus is on the DSSP, which should be able to bootstrap itself and provide services with no human intervention; a pay-as-you-go fashion is cited as a business model. In [10], opportunities for the incremental refinement of used data integration strategies are considered, and the pay-as-you-go approach is cited again. In [11], data integration is described as agile,

Table 1: Comparison among data management approaches cited in this work.

Name	Description	Centralised (C)/ Decentralised (D)	Structured (S)/ Unstructured (U)
relational database	structured collection of data	C	S
non-relational database	organized collection of data	C+D	U
data warehouse	central repositories of integrated data	C	S
data lake	centralised repository to store and process large amounts of structured, semi-structured, and unstructured data	C	S+U
spatial data infrastructure	data infrastructure for spatial data utilisation	C+D	S
data space	federation of decentralised data ecosystems	D	S+U
data mesh	domain-oriented decentralised architecture	D	S+U

and much lower upfront and maintenance costs are foreseen in comparison with Data Base Management Systems (DBMSs). Furthermore, formats and interfaces (relational, sequential, XML, RDF, etc.) are cited, and the accent is put on the fact that data space actors do not have full control over data (which stay under the control of the source), proceeding to integration only whether and when necessary.

The vision behind data spaces comes from the realisation that modern data management scenarios rarely fall into the case in which data can be fit into a conventional DBMS or a single data model. Because of that, the unwritten law of *schema first, data later* used in DBMSs evolves into a novel model of *data first, schema later or never* [3] that data spaces embed at the very core. Figure 1 logically depicts the limits that DBMSs show when it comes to data management scenarios with heterogeneous, differently structured, and unstructured data stored in several independent locations. In other words, DBMSs provide clear benefits when stored data are frequently used, obeying carefully designed schemas, and the data model is subject to very limited changes over time; because of those reasons, the data model receives careful attention from data modellers in the design phase. On the other hand, data more rarely used are more likely to be stored in an unstructured fashion according to [12]. A data space is domain-agnostic (from

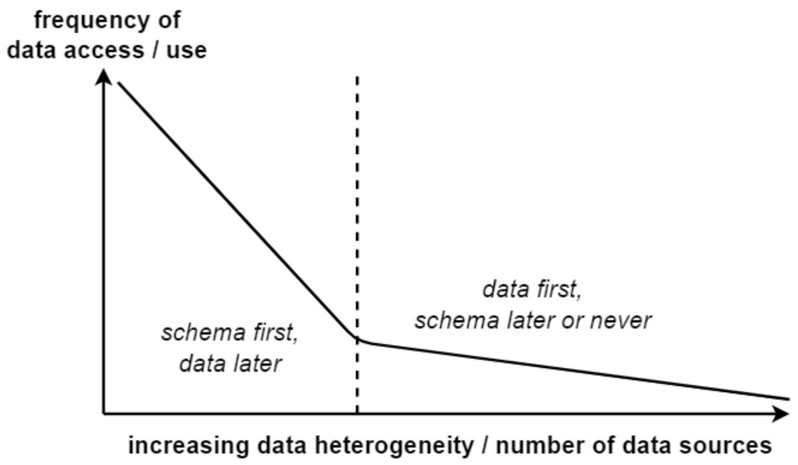

Figure 1: Data spaces as a data management approach also considering the *data first, schema later or never* models (inspired by [12]).

the technological viewpoint), thus it does not foresee the use of a unifying schema for data, proceeding to data integration on an as-needed basis only [3]; furthermore, relationships between resources are to be inferred, not designed [13]. Another key aspect to underline is that querying operations to a data space follow varying service levels, meaning that best-effort or approximate answers may be returned [1]. It translates into producing the best possible results using the available data sources at that time. In fact, a data source can be available or unavailable at any moment because of different reasons, thus its state must be taken into account when performing queries. Such flexibility comes at the cost of a more complicated procedure than that used in DBs. Queries to DBs typically require familiarity with the structure, semantics, and capabilities of the information sources, thus the complexity is handled by the query performer; instead, queries to data spaces have a more unpredictable behaviour and are run in an unfamiliar data environment because of the absence of a rigid centralised management system. To provide answers to queries, a data space relies on a broker to share its inventory of resources and related information, such as source, name, location, size, creation date, owner, and so on. The resulting catalogue of products (data and services) is a key functionality of data spaces [1].

Reference [6] underlines again that a key challenge of modern information management systems comes from having to consider a large number of

diverse and interrelated data sources but with limited possibilities of managing them in a convenient, integrated, or principled fashion. Data spaces are thus *a way to make data co-exist together rather than being integrated*, offering functionalities over data independently from integration. According to a pay-as-you-go model, more sophisticated operations -nowadays common in e.g., cloud-based services- are possible. Actors, for instance users or administrators of the system, can decide where and when it is worth investing more effort in identifying semantic relationships [14], thus focusing on offering services on data rather than guaranteeing *ACID* (Atomicity, Consistency, Isolation, and Durability) properties, which can be guaranteed at the local level if needed. As reported in [13], no dominant proposal or reference architecture had emerged yet in 2009; the authors point out how the lack of an integration schema makes it difficult to evaluate query results because uncertainty on the mapping of different meanings of data in different sources makes it rather challenging to design any automated procedure in this regard.

Before looking at more recent developments when it comes to data spaces, we briefly describe Spatial Data Infrastructures (SDIs) and their role in paving the road for data spaces. SDIs are a comprehensive framework for data infrastructures integrating geographic data, metadata, users and tools to enable efficient spatial data utilisation. They are designed and developed to coordinate agreements on standards and policies, facilitating the discovery and use of geospatial information by users. They serve as the structure for acquiring, processing, and using spatial data. A comprehensive how-to for describing and organizing geospatial data through the use of spatial data infrastructure is given in [15]. Take as reference the European geospatial data landscape, which has been deeply transformed by actions such as Copernicus[1], INSPIRE[2], and the Open Data Directive[3]. Focusing on INSPIRE, introduced in 2004, we acknowledge its large impacts, resulting in the publication of more than 90,000 geospatial datasets in a publicly available fashion. These spatial data sets are uniformly documented and easy to use by members (and not) of the GIS community [16]. In the INSPIRE vision, the primary objective is supporting data-driven decision-making rather than data sharing per se, also focusing on making available data from the pub-

[1]`https://www.copernicus.eu/en`
[2]`https://knowledge-base.inspire.ec.europa.eu/index_en`
[3]`https://eur-lex.europa.eu/eli/dir/2019/1024/oj`

lic sector. However, challenges about incorporating emerging technological trends, user relationships, and socioeconomic factors are still present [17].

SDIs have evolved over two generations. The first generation was driven by data availability, and the second has seen data utilisation and user needs as the driving focus for development [18]. This evolution has yielded several benefits, including an increasing adoption of open data and data sharing, improved efficiency on a national level, promotion of open-source technology and the development of a strong user community. Despite these achievements, several challenges persist. In fact, one issue is the lack of uniform data accessibility, e.g., across EU countries. In fact, only part of the data sets are fully accessible and harmonised, with many still having unclear usage terms or technical content-related issues. SDIs are also somewhat limited by their focus on public sector providers, the rigid encoding of technical aspects in legislation, complex specifications, different parallel implementations (i.e., 'silo' approach), and a lack of user analytics. Another limitation of SDIs is the lack of strategies to access datasets that are considered sensitive or that are not classified as open. The debate around the question of whether SDIs are still needed or should be considered partially obsolete -due to the evolution of the socio-economic and technological context- is still open [16]. However, the exponential growth of data and the increasing heterogeneity of data sources reinforces the need for a robust data infrastructure that meets user needs and requirements [19]. In response to these challenges, data spaces have been proposed.

Similarly to SDIs, data spaces are distributed architectures integrating heterogeneous data sources. A contributing organisation can host data on-premise or in the cloud, with secure access managed through a *data space connector*, which will be presented in the following of this paper. Data spaces offer several advantages over traditional SDIs. They have a stronger push for interoperability, allowing for data integration and harmonisation when needed. They also propose strategies to address the need to provide access to previously inaccessible data through well-documented processes. Additionally, data spaces establish secure data sovereignty processes and offer flexible "pay as you go" usage models. From a policy perspective, a data space represents a distributed system governed by a framework that enables secure and trustworthy data transactions while maintaining data sovereignty.

Looking at recent developments in the field of data spaces, we can start again with definitions, especially from the one proposed by the Big Data Value Association (BDVA) stating that a data space must be considered as

an umbrella term encompassing "any ecosystem of data models, datasets, ontologies, data sharing contracts, and specialised management services (i.e., as often provided by data centres, stores, repositories, individually, or within a 'data lake'), together with soft competencies around it (i.e., governance, social interactions, business processes)" [20]. Data spaces foresee the federation of decentralised data ecosystems based on the use of interoperable software components to interconnect data without relying on any central authorities. The key objective is the discovery of data relations rather than their management, which could be translated into having a common understanding of data instead of a common structure [21].

Building on the recent literature on the topic, which we systematically survey in Section 2, and ongoing initiatives in this field, this work explores the numerous actions that have flourished in the latest years as evidence of the growing interest in the concept of data space and the potential value it holds, especially for the data market. A reference initiative is surely the SOLID (SOcial LInked Data) project [22]. What is relevant in this context is the principle stating that *users' data are managed independently of the applications that create and consume this data*, a principle implemented in the SOLID protocol, in line with the W3 Verifiable Credentials Data Model [23]. Approximately in the same period, the IDS (International Data Spaces) initiative was launched in Germany. Later next year, in 2016, the IDSA (International Data Spaces Association) was founded, and the DSBA (Data Space Business Alliance) followed in late 2021. DSBA puts together the GAIA-X European Association for Data and Cloud AISBL, which was founded in 2019 in Germany to design and develop a federated open data infrastructure; BDVA, creating an innovation ecosystem to enable a data-driven digital transformation; the FIWARE foundation, which provides a framework of open-source software components, data spaces included [24, 25]; and IDSA. To conclude this brief overview of initiatives, we highlight the Data Society Alliance (DSA) founded in 2021 in Japan, the DSSC (Data Spaces Support Centre) initiative that kicked off in October 2022, and the ongoing development of a smart platform at EU level, namely SIMPL [26], since early 2024, to further support the development, testing, and use of data spaces through an open-source middleware solution.

There is a widespread effort to make data spaces available and well functioning because of the key advantages provided by such an approach, i.e., accessibility and reusability of data as well as the adherence to the FAIR (Findability, Accessibility, Interoperability, and Reuse) principles. As evi-

dent by the aforementioned initiatives, a strong interest can be recognised, often backed by institutional and national strategies. The latter ones have a strong interest in a *sovereign* exchange of data, which means being able to self-determine who, how, when, and at what price others may use data across the value chain.

The main objective of this work is to present the current state of technical development and a systematic survey of the literature in recent years on the topic in order to shed light on the current state of development and highlight open issues worth investigating. We base the contents of this work on three questions, as follows: i) what are data spaces?; ii) how do data spaces work?; iii) what components are presently available and what are the expected developments in the future?

This work is structured as follows. In Section 2, we describe the PRISMA methodology used to select relevant scientific works, published in years 2018 to 2023, to carry out a systematic literature survey. Then we present and discuss such results in Section 3. In Section 4, we focus on real-world applications of data spaces to present their uptake in different sectors. In Section 5, we focus on the IDS specifications and analyse the proposed architecture, and in doing so we provide a tutorial-like guide on data spaces, how they work and what the main elements composing them are. Furthermore, we provide pointers for building, running, and testing data spaces using open-source components publicly available. In Section 6, we conclude the work by discussing the current state of development, testing, and use, and by highlighting the still open challenges.

2. Systematic survey

To get clearer insights into the strengths and limitations of existing literature in the field of data spaces, we follow the Preferred Reporting Items for Systematic Reviews and Meta-Analyses (PRISMA) approach [27], facilitating the systematic categorization of works based on their methodological rigour and reporting quality.

2.1. Methodology

This section outlines the criteria for eligibility, the research keywords, the process of identifying records, screening search results, and the analysis approach we utilised to implement this methodology. Figure 2 summarises

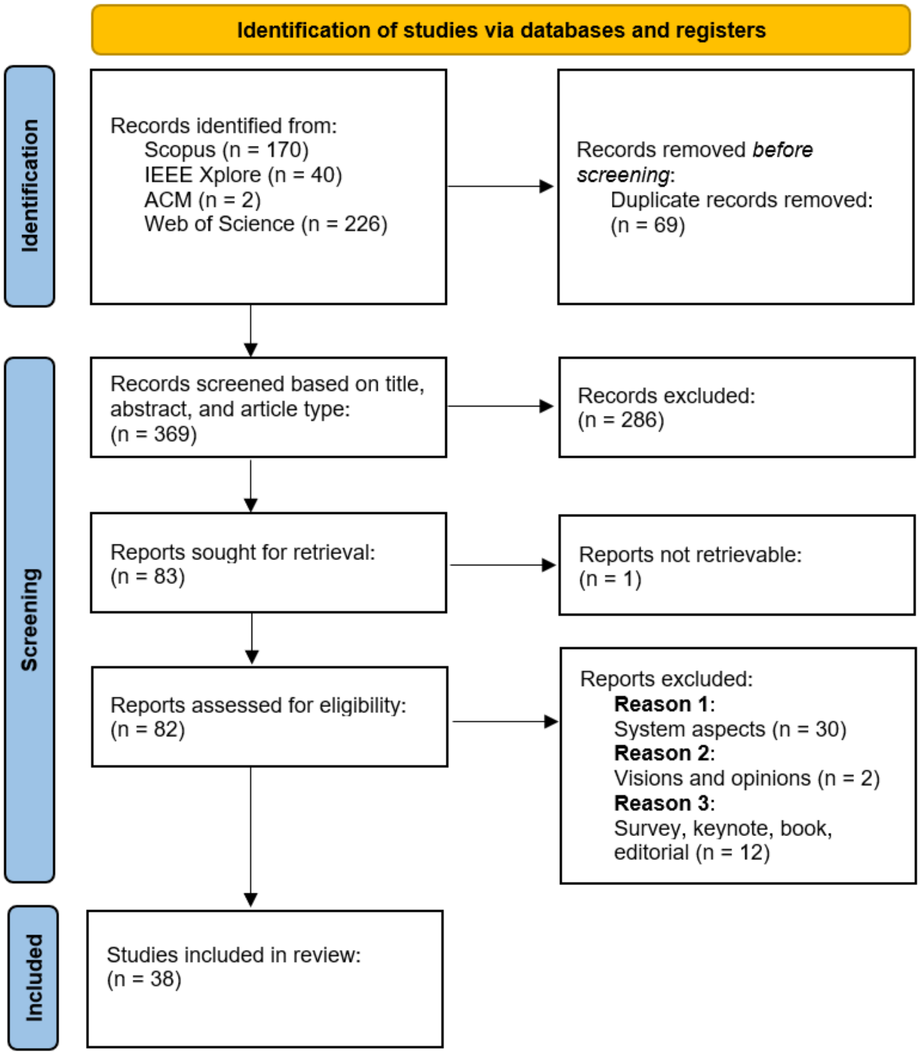

Figure 2: Flowchart of the PRISMA selection process [27].

the PRISMA-compliant search process we conducted, which is fully presented below. The following questions have guided our search in the literature:

- **Q1: Scope and Focus**. What is the scope of the scientific works (vertical vs horizontal, i.e., sectorial vs general approach)?
- **Q2: Data Access Rights**. What are the rights for data use in the considered works? Possible cases are: personal data; public data (ac-

cessed through freedom of information requests); open data available for everyone to access, use and share; and proprietary data with restricted redistribution rights.

- **Q3: Interoperability and Standards**. What are the current challenges and solutions for achieving interoperability between different data sources and platforms in data spaces? What standards (data formats, protocols, metadata) are most widely adopted in data space research, and how do they impact data integration and usage?

- **Q4: Privacy and Ethical Considerations**. How do the scientific works deal with compliance with regulations, such as the European General Data Protection Regulation (GDPR), in the context of data spaces? What ethical considerations are highlighted in the collection, use, and sharing of data in data spaces?

- **Q5: Technological Infrastructures**. What are the key ICT technologies and infrastructure components that are essential for supporting data spaces?

For the eligibility criteria, we consider works that make contributions to the development of data spaces from the system point of view. For the research keywords, we identified records in the title that contain the keyword *data space* or *dataspace* to capture a wide range of research papers. We consider articles published in the years 2018-2023, thus taking into account the most recent developments.

In the identification stage, we utilised four electronic databases, namely "Scopus", "IEEE Xplore", "ACM", and "Web of Science". Based on the searches conducted in the online databases, 438 records were identified (Scopus = 170, IEEE Xplore = 40, ACM = 2, WebOfScience = 226). The documents with the corresponding document identifier (DOI) were then retrieved from the databases. At this stage, 69 duplicates were excluded from the search with Scopus as a baseline, leaving 369 records (Scopus = 170, IEEE = 2, ACM = 2, WebOfScience = 195) documented in the supplementary material. We analyse the identified records by subject area (Fig. 3a) and by year (Fig. 3b). Regarding the former, it can be seen that more than half of the publications are in the field of Computer Science followed by a substantial focus on engineering topics (17 %) and moderate interest in Earth

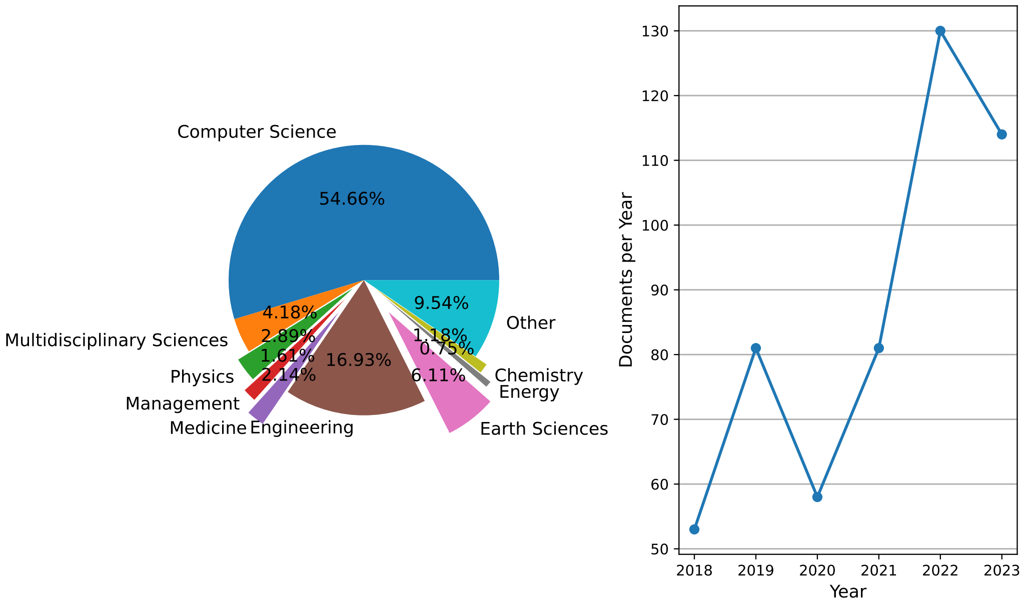

a) Documents by Subject Area b) Documents per Year

Figure 3: Analysis of the 369 identified records in the PRISMA procedure.

Sciences (6 %). Looking at the number of documents published per year, the pattern shows an increasing activity over time, despite some fluctuations.

We now describe the two screening stages we carried out to select the most relevant works. In the first screening stage, the title and the abstract of the papers selected in the identification stage have been analysed. Recalling that our interest is in works providing a system view of data spaces, we excluded not relevant papers according to the following Exclusion Criteria (EC):

- **EC-1**: the publication does not discuss data spaces from a system or architectural design viewpoint (e.g., it focuses on a specific and narrow aspect of the design only);
- **EC-2**: the publication offers qualitative visions and opinions only;
- **EC-3**: the publication is a survey, a literature review, a book, a keynote or an editorial.

This stage eliminated 286 records, thus leaving 83 papers for further examination (Scopus = 75, IEEE = 1, ACM = 1, WebOfScience = 6). In the

second screening stage, we conducted a more thorough examination of the 83 works, and finally selected the studies meeting the following inclusion criterion (IC):

- **IC**: the work focuses on the comprehensive definition and development of the data space system.

In this phase, we additionally excluded 30 works because the IC was not met. Moreover, 2 works are visions and opinions (not recognisable in the first phase), and 12 have the form of a tutorial or keynote, which was not possible to identify as such beforehand either. Finally, one work is not accessible. In conclusion, we selected 38 records (38 from Scopus and 1 from ACM) for a more in-depth study, fully described in Section 3.

2.2. Novelty

In this section, we highlight the novelty of our approach with respect to other reviews identified in our SLR (see supplementary material). Two are the results that stand out, specifically [28] and [29].

In the field of Industry 4.0, the data space concept is explicitly mentioned in [28]. Using a PRISMA-based approach for the review, the authors offer an interesting perspective on the concept of data ecosystem, highlighting the need for vertical and horizontal interconnectivity across the entire chain. Given the increasing importance of standardized data models and data-sharing initiatives, they consider the Reference Architectural Model Industrie 4.0 (RAMI4.0) [30] as most relevant in the field and explore its relationship with the IDS model we describe in Section 5. In our work, we adopt a more general approach offering a historical view of the evolution of data spaces and considering the general architecture with no specific focus on given use cases. Additionally, we provide pointers to existing software components (see Section 5.4), and describe real-world applications already using the data space approach (see Section 4).

In [29], the authors consider Earth Observation and the role of SDI platforms. As anticipated in Section 1, SDIs are capable of managing, storing, processing, and analyzing large geospatial data sets. Dating back to the last years of the past century, SDIs have evolved by integrating novel technologies over time and growing in both size and offered functionalities. The authors compare several existing platforms, showing different system architectures

and analysing their features in terms of data, processing, infrastructure abstraction, open governance, reproducibility, replicability, scalability, data access interoperability, and extensibility. Anyway, the work does not explicitly consider data spaces and focuses on the specific domain of geospatial data. As anticipated above, we adopt in this work a more general approach, analysing and discussing the key characteristics of data spaces and their adaptability to different use cases.

3. Systematic Literature Survey: Results and Discussion

The 38 selected publications, classified according to the 5 questions in Section 2.1, are presented in Table 2.

3.1. Scope and Focus

We found that most works aim at specific sectors vertically, such as agriculture, finance, healthcare, and manufacturing among others, and some of them horizontally span various industries and domains. Focusing on the former, we have identified the following sectors:

- Agriculture: [53, 54, 63]
- Energy sector: [47]
- Healthcare: [46, 59]
- Smart cities: [33, 67]
- Manufacturing: [24, 45, 48, 57, 39, 62]
- Maritime operations: [38, 42]
- Search engine for researchers in academia: [35]
- Automated Guided Vehicle: [49]
- Environment monitoring: [61]

Other works follow a horizontal approach to data spaces covering various industries and domains.

Ref.	Year	Scope	Data Type	Interop.	Privacy/Ethics	Technology
[31]	2018	Industrial (H)			GDPR	
[24]	2018	Manufacturing (V)	proprietary			FIWARE
[32]	2018	Industrial (H)				Android
[33]	2019	Smart Cities (V)		intra-DS		
[34]	2019	IDS (H)	public		anonymisation	Spark, Kafka, SQL
[35]	2019	Academia (V)	open			Protégé
[36]	2019			intra-DS		
[37]	2019			inter-DS	GDPR	
[38]	2019	Maritime (V)	proprietary		GDPR	FIWARE
[39]	2019	Manufacturing (V)			GDPR	
[40]	2019				3-layer security	
[41]	2020	IDS (H)			GDPR	
[42]	2020	Maritime (V)	proprietary	inter-DS	GDPR	FIWARE
[43]	2020	IDS (H)		inter-DS	GDPR	FROST
[44]	2020	IDS (H)		inter-DS	GDPR	
[45]	2021	Manufacturing (V)				
[46]	2021	Healthcare (V)			GDPR	
[47]	2021	Energy sector (V)				
[48]	2021	Manufacturing (V)				
[49]	2021	Autom. Vehicle (V)	open (GitHub)	inter-DS	GDPR	Kotlin app with Docker
[50]	2021				GDPR	
[51]	2021	IDS (H)			GDPR	
[52]	2021	Time-aware (H)				ELK stack
[53, 54]	2022	Agriculture (V)		inter-DS	GDPR	
[55, 56]	2022	IDS (H)		inter-DS	GDPR	Triplestore, Mendix
[57]	2022	Manufacturing (V)				
[58]	2022	IDS (H)		inter-DS	GDPR	Java
[59]	2022	Healthcare (V)		inter-DS	GDPR	
[60]	2022			inter-DS		
[61]	2022	Environment (V)	public			Docker,Kubernetes,several DBs,Linux
[62]	2022	Manufacturing (V)				
[63]	2023	Agriculture (V)		inter-DS	ethical use of data	
[64]	2023		open (GitHub)		federated learning	
[65]	2023	Solid (H)		intra-DS		
[66]	2023	Industrial (H)		inter-DS		
[67]	2023	Smart Cities (V)				

Table 2: Research papers related to data spaces ranging from 2018-2023. The scope can be horizontal (H) or vertical (V).

- A data connector store compliant with the IDS specifications (discussed in Section 5) is designed in [55, 43, 58], and a Policy-Agnostic Programming Language can be found in [41]. A real-time linked data space for Internet of Things (IoT) for various domains is proposed in [34]. A reference enterprise architecture is outlined in [56]. Finally, [51] presents a set of methods for the development of user-centred interaction design patterns.

- For the Industrial Data Space, bridging the gap between IDS and Industry 4.0, the works in [66] elaborate on the key design options of data, services, and computing infrastructures. The paper in [31] performs a model-based privacy analysis to enable the verification of conformance to customer's privacy preferences. Requirements for key security requirements in untrusted environments are outlined in [32].

- A social database that is built on top of the (Semantic) web and Solid is addressed in [65].

- In connection with IoT, [52] proposes time-aware data spaces.

3.2. Data type

Another classification of data spaces is the types of data involved, such as personal data, public data, open data, or proprietary data. The origin of data may arise from public sources, private enterprises, research institutions, or a combination of those.

It is worth pointing out that only very few works present experiments with data. Among them, reference [24] uses proprietary data of unknown origin to validate the industrial IoT data space; reference [34] proposes a data space that links data from various (publicly available) data sources; reference [64] uses a predictive maintenance open dataset -publicly available on GitHub- from one of Schwan's factories. Reference [49] exchanges IDS messages in an automated guided vehicle use case using an open dataset available on GitHub, as well. The industrial data space architecture for the seaport of Valencia, Spain, has been tested with proprietary industrial data of private companies [38, 42]. To share tables, XML, and images among research institutions, the research data space described in [35] contains approximately 600 hundred open records (at the time of publication). Finally, we present the publicly available dataset in [61] as part of the Green, Adaptive and Trustworthy Edge (GATE) Urban Data Space project, which we further explore in Section 4.

In this work, IoT-generated air quality data from various sources are used to feed a data space to provide an easy-to-use, secure and reliable data sharing for the authorities, business partners as well as citizens.

3.3. Interoperability

Discussing the level of interoperability and compatibility between different data sources, platforms, and systems, we need to distinguish between two models: intra-data space, where various connectors from different participants operate within a single data space; and inter-data space, involving connector protocols for interoperability among different data spaces. Note that a data space alone does not represent its participants but facilitates trust and data sharing among its participants.

Concerning approaches towards inter-data space exchanges, we point out the work in [59] discussing interoperability requirements of the health records to build the European Health Data Space (EHDS). To ensure syntactic interoperability, the European Commission will follow recommendations to create a European Electronic Health Record Exchange Format. To address technical interoperability between the Member States, the EHDS will further develop a cross-border digital infrastructure in the form of a central platform, referred to as the e-Health Digital Service Infrastructure. The work in [60] addresses the identification of such harmonisation profiles in the context of IDS specifications. In the meantime, a growing number of connectors make data sharing between enterprises increasingly appealing [56, 58], especially in connection with IoT devices [43, 49]. Within this context, the study in [37] focuses on early platform design phases. Most recently, a few interoperable data spaces compliant with IDS specifications have been realized. For example, the agricultural DS in [54] allows for data sharing between service providers at both syntactic and semantic levels. Reference [63] presents a reference architecture to enable interoperability and data sovereignty in the Agricultural Data Space. For the industrial data space, reference [66] presents a layered architecture design including a federation layer that supports data, service, and computing infrastructure interoperability. A noteworthy use case for the industrial data space is the seaport scenario in [42], which presents the implementation details and carried-out tests of the architecture as a solution to overcome the data interoperability among the stakeholders shipping company, port authority and terminal operator. To enable interoperability between a variety of data spaces, a set of harmonisation profiles is required in the harmonisation domain to provide the necessary functionalities.

So far we have considered inter-space interoperability. Let us now consider intra-space interoperability. In the literature, IDS-based cases are intended for companies and industry use cases, while the "Solid Data Space" [22], a concept for data spaces that build on top of the (Semantic) Web and Social Linked Data (Solid), aims at people in control of their personal (interested readers are referred to [68] for additional insights). Following this approach, the study in [65] discusses interoperability at various layers and the "data space layer" offers interoperability among applications. Furthermore, we want to point out the work in [33] tackling intra-space interoperability ranging from switches, routers, and media gateways, to end-user terminals such as smartphones, tablets, and desktops from various manufacturers for urban data space. A data space platform that supports verifying compatibility (and eventually summarisability) of the underlying data is available in [36].

3.4. Privacy and ethical considerations

Let us now focus on how existing works tackle data privacy as well as ethical guidelines or principles governing the collection, use and sharing of data within the data space. Adhering to ethical data principles involves implementing strong data security measures to safeguard against breaches and unauthorized entry.

We begin with the topic of data privacy. The European Union takes data privacy seriously and therefore adopted the General Data Protection Regulation (GDPR) in 2016. Compliant with this approach and thus supported by the European Union, the FIWARE platform is a valuable middleware solution for the deployment of IoT applications. A FIWARE-based implementation of the industrial data space architecture can be found in [24, 38, 42]. The components are open source, and FIWARE processes personal data complying with GDPR. The GDPR-compliant Trusted Integrated Knowledge Data Space (TIKD) in [46] is an Irish commercialisation approach to securely share data in collaborative environments by considering personal data handling, data privileges, access control context specification, and a privacy-aware data interlinking. The European Commission is working on a European Health Data Space enabling citizens of the European Union to gain secure access to their electronic health data according to GDPR [59]. Finally, it is worth pointing out the industrial data space [31] as one of the first initiatives to create and use smart IT services, then followed by the

IDS-compliant initiatives [37, 39, 43, 50, 51, 49, 55, 56, 54, 58, 41] at European/global level, ensuring data privacy and data protection according to GDPR.

Other data spaces have a more limited privacy protection. Specifically, the real-time linked data space for IoT-enabled smart environments by [34] anonymises user data. The ”Authentic Data Space” architecture proposed in [40] incorporates a hierarchical data security model comprising four security layers: grading, marking, technology and control. The Agricultural Data Space proposed in [63] explicitly requires that all participants agree on the use of an open shared vocabulary providing the data with semantics.

In contrast, reference [64] follows a federated learning approach that supports privacy-preserving distributed collaboration based on the GAIA-X framework [69]. Data sources can establish a federation to allow the training of models on their data, which remain at the source and are not available at the orchestrator. What is shared are the parameters of the models trained on the data at each source. Note that data spaces for sensing, storing and managing environmental data are of public interest and do not follow privacy policies intentionally [61].

3.5. Technological infrastructure

Let us identify key technologies and infrastructure supporting data spaces. Most of the identified papers propose theoretical concepts. A few, however, come up with proof of concepts. Alonso *et al.* present in [24] a prototype of the IDS architecture implemented in FIWARE that has been validated in a real industry case. Reference [38, 42], in contrast, proposes a FIWARE IoT platform that implements the IDS architecture for improving logistic maritime operations in a seaport scenario; a use-case implementation has been realized in the Valencia port, Spain. To interface IoT with IDS, reference [43] prototypes an IDS connector that uses the Fraunhofer Open Source SensorThings (FROST) API Server -including a database, a web interface, a Representational state transfer (REST) and an MQTT (Message Queuing Telemetry Transport) interface- to transmit, store, and query sensor data. Reference [49], in contrast, develops an IoT-compatible IDS test environment. An open-source Kotlin application using Docker containers has been developed to emulate two popular schemes (request/response and publish/subscribe) for sovereign IoT cloud communication as well as to simulate an automated guided vehicle (AGV) use case. The IDS connector store described in [55] for a broker service is built upon a triplestore (RDF) database.

The front end is a web application developed in Mendix. The IDS connector reference implementation in [58] is a stateless REST server composed of multiple APIs in Java. Finally, we cite the work in [32] as architecture to build trust and security -leveraging containerisation- running isolated packages on a single Android device.

An open cloud virtual data space model, based on the Web Ontology Language (OWL), and using the open-source ontology editor Protégé, can be found in [35]. A real-time data space for IoT-enabled smart environment based on a "pay-as-you-go" (open-source) CKAN-based data space catalogue is available in [34], using Apache technology like SQL, the Kafka message broker, Spark streaming, and Druid real-time analytics. The proposed framework has been tested in five real-world smart environments in the European Union. Reference [52] prototypes a time-aware data space using the open-source Elasticsearch, Logstash, and Kibana (ELK) stack. It supports all distributed search and analysis engines for several types of data. To monitor air pollution, Vassilev *et al.* show in [61] a prototype of a cloud-based data platform operating under Linux and controlled by Kubernetes container management system. Various databases like Postgres, MongoDB, and Neo4J, using the exchange format 3DCityGML, are deployed within Docker containers using YAML scripts.

4. Real-World Applications of Data Spaces

In this section, we describe a few implementations of the data space approach as used in real scenarios. Two come from the literature, specifically the GATE project [61] and the Fraunhofer lighthouse project "Cognitive Agriculture" (COGNAC) [54], and one is the output of a large effort in the agricultural sector in Belgium, France, and Finland, giving rise to separate data space initiatives that merged into a transnational agrifood data space in 2024.

We start with the GATE Urban Data Space project, sharing data and services covering two use cases: the air quality prediction, and the social facilities coverage. The former provides real-time prediction of air quality in the city of Sofia, Bulgaria; the latter provides data to decision-makers to evaluate the coverage of social facilities (e.g., schools, hospitals, clinics, meeting points, etc.) in the city neighbourhoods. The Data Platform is a holistic ecosystem that covers the entire data value chain, including data management, analysis, and visualization. It integrates industrial Big Data frameworks (such as

Oracle, IBM, SAP, and Bosch) with open-source software (like Hadoop and Kubernetes). The platform adheres to open standards, ensuring interoperability and acting as a gateway to other EU infrastructures like FIWARE, EGI, and BDVA i-Spaces [61].

Let us focus now on the COGNAC Agricultural Data Space (ADS), a Fraunhofer lighthouse initiative, aiming to facilitate the availability of environment, operational, machinery, and process data in digital farms. Based on the IDS conceptual approach, which we explore in Section 5, it has the objective of connecting different already existing solutions seamlessly to support decision-making along the agricultural value chain. COGNAG focuses on maximising productivity and sustainability. Key components of the ADS are, according to [54], sensing units to record both agricultural operations and environmental data, software tools for data evaluation and analysis to derive insights, then fed into Decision Support Systems (DSS) for fieldwork and animal husbandry. In [54], the authors describe three scenarios, sustainable management of the nutrient cycle, business models and legal obligations, and agricultural e-government. The last one is a critical scenario at present, especially from the point of view of alleviating the administrative burden on farmers

Finally, we consider another example, again in the agricultural field. Several initiatives have flourished over the years, such as the agricultural and agri-food data intermediation platform AgDataHub in France dating back to 2015; the Agricultural Datasharing Platform DJustConnect in Flanders, Belgium; the Agrifood Data Space in Finland; the JoinData platform in the Netherlands; the DKE agrirouter in Germany; and the Agrimetrics in the UK. What is important about those initiatives is that they share the willingness to facilitate data sharing and interoperability in agriculture, with clear business models, and often focusing also on easing the farmers' administrative burden, as anticipated above. The vision behind data spaces sees different data-sharing initiatives, like the ones above, connected to each other to form a network. In mid 2024, the first transnational agrifood data space was announced, joining together three platforms, DJustConnect, AgDataHub, and Tritom from Finland. The first case of real-world cooperation among the three platforms revolves around the farming sector, specifically the case of potatoes in the Potato-X initiative [70].

We conclude by highlighting that AgDataHub is registered as a data

intermediation service[4] as specified by the European Data Governance Act. This, and other aspects, are covered in detail in Section 5, discussing how such a variety of approaches and deployments are going towards a single set of specifications.

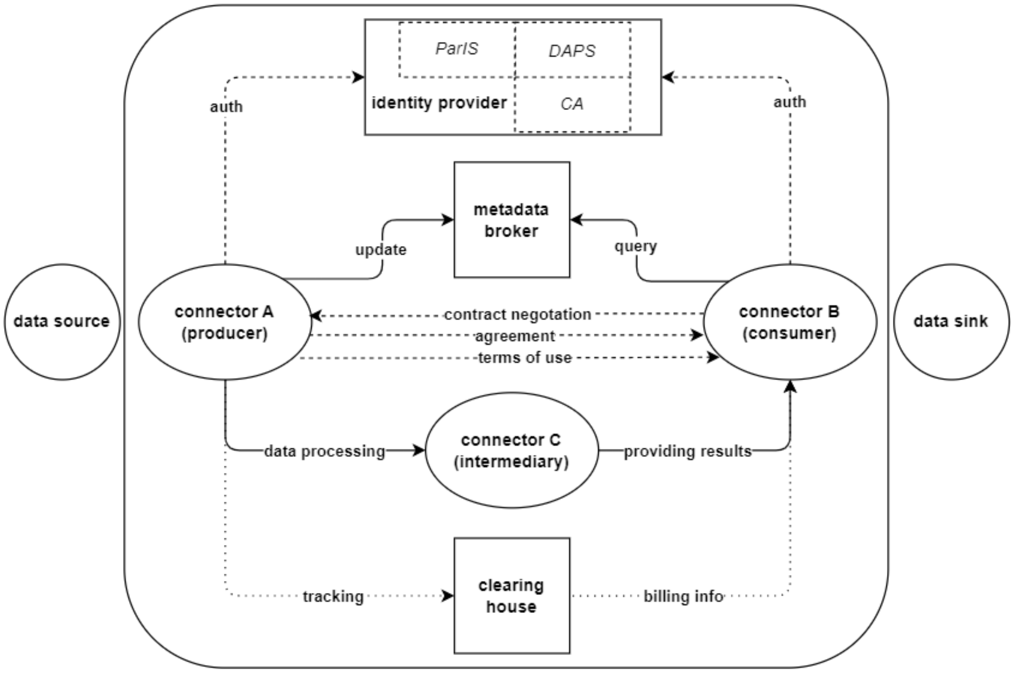

Figure 4: Interactions among the main technical components in a data space. Dotted lines are for control and administrative messages, and solid lines are for data and metadata exchanges.

5. Toward Common Specifications for Data Spaces: the IDS Reference Architectural Model

In this part, we focus our attention on the IDS Reference Architecture Model (RAM) [71] on the one hand, and practical aspects on the other hand, presenting the main elements composing a data space, the minimum set of

[4] https://digital-strategy.ec.europa.eu/en/policies/data-intermediary-services

components needed to run it, roles and actors, and existing guides to build the components.

Similarly to data meshes [72], data spaces are a federation of existing entities in the form of products, producers, and consumers. Products can be datasets or applications to be exchanged or run, respectively. The product description follows the specifications defined in the W3C Data Catalog Vocabulary (DCAT) [73]. Note that there are specialisations of the IDS RAM being developed, such as the Reference Architecture Model Industrie 4.0 (RAMI 4.0) [30] in the context of the Industry 4.0 paradigm. RAMI 4.0 or other proposals will not be discussed in this work, we keep our focus on the general model.

In what follows, Section 5.1 focuses on the building blocks used to set up data spaces. In Section 5.2, our attention focuses on the several actors and related roles foreseen in the ecosystem. Then, in Section 5.3, we survey the available implementations of connectors, i.e., the key element to be deployed at each endpoint. Finally, Section 5.4 provides pointers to guides and tutorials to test and run data spaces and composing elements.

5.1. Technical Components of a Data Space

In Section 1, we presented a short survey of how the definition of a data space has evolved in the last 20 years. Then, in Section 2, we surveyed the scientific literature on this topic, showing how the concept is penetrating different sectors. We now look at both main and support technical components available at today, which can be used to build data spaces according to the IDS RAM. The main components and related message flows are depicted in Figure 4, and are presented in detail below.

Main Flows and Components

With data spaces being federated decentralised ecosystems, components must be deployed at each data source. This is the task of what should be considered as the key component, namely the *connector* (participating agent). Simply put, the connector *establishes the connection to the data sources of the data providers, manages metadata of the data sources and the terms of use of the data and sends or receives the data, including the terms of use* [74, 75]. Data exchanges follow the specification defined in the dataspace protocol (DSP) [76], which is a set of specifications designed for interoperable data sharing among different entities, relying on Web-based technologies. The dataspace

protocol represents the de facto standard[5], which is implemented in several different versions of the *connector* component, described in Section 5.3. The connector follows the philosophy of decoupling the control plane from the data plane, as common in Software-Defined Networking deployments.

In addition to the connector, another main component is the *identity provider*, which is in charge of maintaining and verifying the identities of the connectors (participants) according to the aforementioned W3 Verifiable Credentials Data Model. In fact, each connector must be authenticated before any operation in the data space. The identity provider is composed of three sub-entities: the *Certification Authority* (CA), the *Participant Information Service* (ParIS), and the *Dynamic Attribute Provisioning Service* (DAPS). The CA is in charge of issuing and revoking identity certificates. The ParIS receives, persists, and shares the connector self-descriptions for other connectors to query and retrieve information about the participants' identities. Finally, the DAPS allows enriching the aforementioned connector self-description with supplementary attributes (such as temporary changes in participants' trustworthiness, information about known vulnerabilities or newer versions of software components in use, and certificate revocation) as well as verifying them. Note that the identity provider may not be needed in pure P2P ecosystems in which no authentication process is foreseen. However, the most common scenarios will use the identity provider to provide trusted data exchanges among identifiable parties.

Once authenticated, each connector can look for data assets in the data space by querying the *metadata broker*. The latter is an intermediary service specialised in providing search functionalities, as well as in sharing relevant information (such as status updates, and newly available data) with other interested (i.e., subscribed) connectors, thus allowing for metadata describing data assets to be available in the ecosystem, according to the PUB/SUB paradigm widely in use nowadays. Once a data asset of interest is identified by a connector acting as a data consumer, it proceeds to ask the other party (connector acting as data producer) its *catalogue*, i.e., shared data or services, as well as their terms of use; the latter is shared in the form of a *policy*. A *contract negotiation* can then start between the two parties to

[5]The Eclipse Foundation is developing a Specification Project to create an ISO standard from version 1.0 of the DSP. Further details at `https://projects.eclipse.org/proposals/eclipse-dataspace-protocol`.

reach an *agreement*. If successful, the *transfer process* can initiate, otherwise the negotiation is terminated. Each transfer process is logged in the *clearing house*, a component keeping track of all the transactions; if billable, the clearing house shares information for settlement with the involved connectors.

Support Components
We describe below the most relevant support components available today. Additional classes of support components may be added to data spaces in custom setups after defining their roles and functions.

The IDS *information model* describes all the basic concepts (such as roles and functions), i.e., how the products are described, published, identified, exchanged, and consumed. The *vocabulary provider* or *vocabulary intermediary* manages ontologies, reference data models, and metadata elements describing data assets. Formally, the aforementioned information model is the low-level vocabulary provider, but additional domain-specific vocabularies may be used in specialised contexts. A *vocabulary hub* service can be used to persist and make accessible multiple domain-specific vocabularies to connectors. The information model, the vocabulary provider, and the domain-specific ontologies form the basis for semantic interoperability, which is not discussed in this work.

The *app store* is in charge of distributing apps, which the requesting connector has to download for use. It is conceptually equivalent to the metadata broker, but brokering apps instead of metadata.

5.2. Main Actors and Roles in a Data Space

In this section, we briefly explore the actors and the roles in a data space, using an example for clarity. Note that Figures 4 and 5 purposely use a different terminology for clarity reasons.

Consider Figure 5: at the very centre there is the data generator and user; in our example a Small and Medium-sized Enterprise (SME) (e.g., a small agricultural farm) who is using IoT devices to collect e.g., soil data. The devices are the *data producers*, sending data to a remote cloud platform (an information system e.g., for soil monitoring) offering services on top of collected and stored data. Thus, the cloud platform is the *data holder*, and the farm is the subject whose data are collected, stored, and processed. *Data*

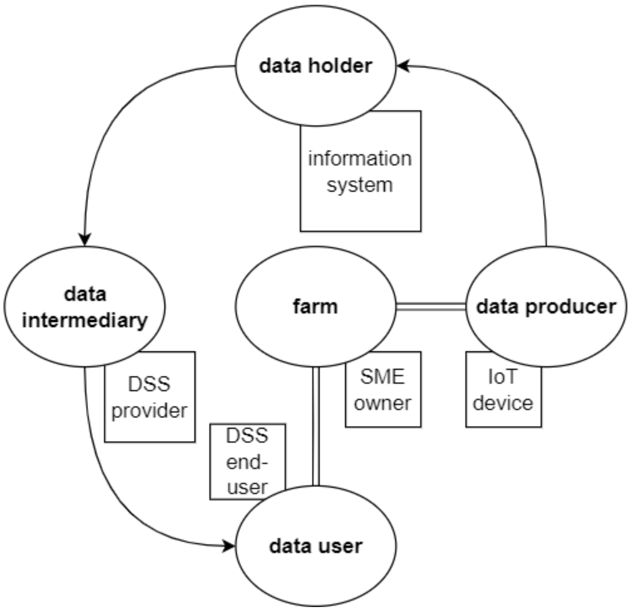

Figure 5: Roles are depicted in rounded blocks. Squared elements provide practical examples of different roles.

intermediaries[6] are represented by those accessing and using data to e.g., provide additional services to *data users*. The latter may be interested in using processed data for their purposes, relying on intermediaries capable of carrying out the needed elaboration. In our case, the intermediary could be the provider of a cloud-based Decision Support System (DSS) used by the farm e.g., for precision agriculture services. The intermediary accesses several data sources, such as micro-climate data from different information systems, to feed their algorithms and estimate plant water needs, thus providing support to the farm, which is the *data user*.

5.3. Data space connectors

The DataSpace Connector released by IDSA [78] was the first connector being released, which served as the reference implementation for its successors. This connector was originally developed at Fraunhofer ISST and is now maintained by Sovity GmbH [79] which offers both free and paid connectors

[6]We point readers to [77] for additional insights on data intermediaries.

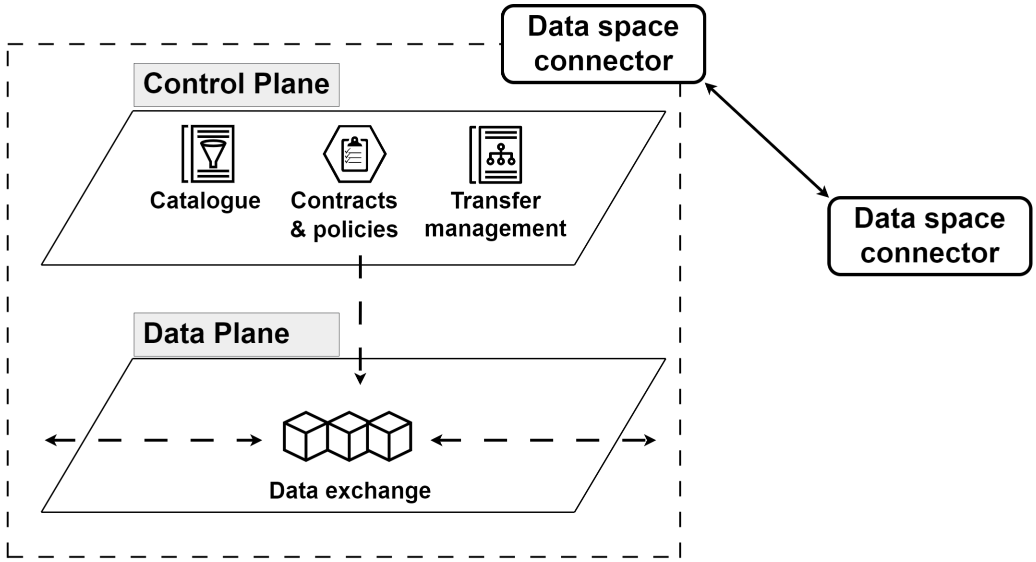

Figure 6: Control plane and data plane in the architecture of the connector.

Table 3: Most mature open-source connector implementations in public repositories.

Name	Repository	TRL
DSC sovity (community ed.)	`https://github.com/sovity/edc-extensions`	9
EDC	`https://github.com/eclipse-edc/Connector`	8-9
TNO Security Gateway	`https://gitlab.com/tno-tsg`	8
OneNet	`https://www.onenet-project.eu/`	7
FIWARE	`https://github.com/FIWARE/data-space-connector`	6-7
TRUSTED	`https://github.com/Fraunhofer-AISEC/trusted-connector`	6-7
TRUE	`https://github.com/International-Data-Spaces-Association/true-connector`	6
GATE	`https://github.com/gate-institute/DataspaceConnector/tree/main`	5

for different purposes. In the IDSA concept, a connector is the core of a data space. It is the gateway to connect systems and data to the data space ecosystem. Its architecture and functionalities are defined by the IDS RAM and specified by the certification criteria available in [80].

The Eclipse Dataspace Connector (EDC) and related components represent the successor to the IDSA one, based on an architecture designed for production-level applications. An overview of these components, with the developer resources, is available online [81]. The EDC can operate independently or be linked to existing back-end systems. Numerous data spaces employ the notion of a connector to establish a connection between a system/organisation and a data space. This connector is associated with different components, such as data exchange, identity management, and usage policies. Each of these functions may be executed on different containers to keep the implementation of the connector as lightweight as possible. In addition, these functions are independent of each other and can be easily replaced using different implementations.

The connectors follow the SDN model, i.e., are logically divided into two parts, shown in Figure 4: the *control plane* and the *data plane*. The control plane handles data management, routing, and processing, while the data plane is responsible for data exchanges. For example, the control plane deals with user identification, the implementation of access and usage policies, whereas the data plane manages data transfers. Thus, in the control part, the connector provides verification, contract negotiation, oversees policy enforcement, and manages provisioning. As a consequence, the control plane relies on common standards to provide the aforementioned services. In contrast, the data plane may depend on different requirements because different types of exchanges (e.g., notifications, messages, events) occur in different scenarios.

In Table 3, we list active open-source projects developing connectors compliant with the IDS RAM and the DSP. We consider implementations showing a Technology Readiness Level (TRL) greater than or equal to 5 (technology validated in relevant environment). The main source of information is the *Data Connector Report* by IDSA [82], in which 30 connector implementations are listed. Amongst them, we focus on open-source connectors, available in public repositories, and being actively maintained.

Table 4: Practical pointers to components publicly available for developing, configuring, and testing data spaces.

#	Description	Link
1	IDS MVDS	`https://docs.internationaldataspaces.org/knowledge-base/mvds`
2	IDS MVDS deployment guidelines	`https://internationaldataspaces.org/how-to-use-the-minimum-viable-data-space-in-the-ids-testbed`
3	Setting up an IDS MVDS	`https://github.com/International-Data-Spaces-Association/IDS-Deployment-Scenarios`
4	EDC MVDS	`https://github.com/eclipse-edc/MinimumViableDataspace`
5	EDC tutorials	`https://github.com/eclipse-edc/Samples`
6	IDS testbed	`https://github.com/International-Data-Spaces-Association/IDS-testbed`
7	IDS specifications v. 0.8	`https://docs.internationaldataspaces.org/ids-knowledgebase/v/dataspace-protocol/overview/readme`
8	IDS Specifications per component	`https://github.com/International-Data-Spaces-Association/IDS-G`
9	Step-by-step guide to build IDS data spaces	`https://github.com/International-Data-Spaces-Association/idsa/tree/main/how-to-build-data-spaces`
10	Deploy of the IDS connector	`https://international-data-spaces-association.github.io/DataspaceConnector/Deployment`
11	FIWARE DSBA-compliant Dataspace	`https://github.com/FIWARE-Ops/fiware-gitops/tree/master/aws/dsba`

5.4. Running and Testing Data Spaces

In the following, we provide practical pointers to interested readers referring to various components publicly available.

1. Minimum Viable Data Space (MVDS)

An MVDS (see #1 in Table 4) can be defined as the minimum set of components to initiate a data space offering features such as secure and sovereign data exchanges. The minimum set of components comprises at least two connectors (one data producer, and one data consumer) and the identity provider. An MVDS can be set up by following the guidelines (see #2 in Table 4) and the scenarios available online (see #3 in Table 4), which we explore below. The first step is providing each participant with a certificate, and one can rely on the pre-configured CA providing default certificates. Then, the DAPS must be set up on a dedicated server; its implementation available in the IDS testbed is pre-configured to accept the aforementioned default certificates. Moving to the connectors, the data provider has to define the metadata, the policy (terms of use) e.g., using a pre-filled standard policy file by IDSA, the representation (e.g., a JSON file and its MIME type), and the catalogue with the resources to be brokered. The data consumer accesses the contract in the metadata and accepts it, thus gaining access to the resources through a designated URL via the connector. Similarly to IDS, the Eclipse Foundation has released an MVDS based on the Eclipse Dataspace Components (EDC) (see #4 in Table 4). Additionally, there are four different tutorials by the Eclipse Foundation available online (see #5 in Table 4) covering how to run a connector, how to transfer data among endpoints, how to collect telemetry-like information about events (e.g., requests, contracts, and data exchanges) in the data space, and how to enforce policies.

2. Compliance with specifications

The IDS testbed (see #6 in Table 4) is designed to verify whether the implementation of a software component meets the IDS specifications (see #7 in Table 4). In this sense, it can be used to check whether any implementation complies with IDS-certified data spaces before deployment. Specifications of each component are available online (see #8 in Table 4).

3. Your Data Space

To build a custom data space, one can use the several open-source components available online, which we surveyed in Section 5.3 (see Table 3), following IDS step-by-step guides (see #9 in Table 4). The connector compliant with the IDS RAM [71] can be quickly deployed (see #10 in Table 4). Finally, we mention the tutorial by FIWARE to set up a DSBA-compliant data space (see #11 in Table 4).

6. Discussion and conclusions

Data spaces are entering into their third phase. In fact, in the first decade of this century, the first wave of data space initiatives [1, 83, 84] have been a sort of large-design competition consisting of proof-of-concept projects that explored the potential for data spaces within specific data management use cases. This phase was followed by a second one, now underway [85, 86], in which the focus is on general deployments of data spaces beyond the specific use cases in order to drive broader adoption. However, with their progressive adoption and convergence towards more mature (almost standard) and working implementations, the seeds for the next phase -that of maturity- are already set. We mention the DSSC blueprint [87] as a major step in terms of maturity. Looking at the close future, we expect that decentralised identity management will become a key asset, while human-centrality, legal, and machine-readability aspects will be at the very core of coming discussions.

There is, however, an inherent risk in this evolution, concerning the trade-off between functionality and complexity. If the initial success of data spaces is motivated by the need for interoperability and data sovereignty, the progressive development of specifications and working implementation results in the incremental growth of meanings, functionalities and expectations which raises the complexity of the current specifications. The risk lies in the progressive loss of the minimum level of service and components necessary to achieve it, which may raise a barrier to their widespread adoption. We stress how both *minimum interoperability mechanisms* and *minimum viable components* are critically needed.

In this context, this work has provided a perspective on the state of the art of data spaces, built around three key questions: what are data spaces nowadays, how do they work, and what are the components presently available and the expected ones in the future. Our answer to the first question is, in a sense, indirect, as we develop a wide-ranging systematic analysis of the state of the art in the scientific literature. The answers to the next two questions are instead more direct. We identify in the IDS specification the emerging architectural model and we align to it in the description of the working model and components organization. We devote an important part of our presentation to the connectors, as they are the access points to data spaces for both developers and stakeholders.

Our vision for the future of data spaces is that they will enable a dynamic global data ecosystem and data markets, where increasing amounts of

data move as goods move nowadays in supply chains. Such revolution will bring challenges with it, as those related to the development of worldwide, trusted data-sharing platforms and mechanisms, usage control, decentralised governance, and business models, crossing other emerging research areas concerning e.g., cognitive adaptability and explainable artificial intelligence. In our ongoing and future work, we are interested in keeping a view on these trends and developments and in exploring their applications to specific fields, as in the case of digital agriculture [88].

Acknowledgements

This work is partially funded by the Horizon Europe CODECS (Maximising the CO-benefits of agricultural Digitalisation through conducive digital ECoSystems) project, GA 101060179, website `https://www.horizoncodecs.eu`; and by the Italian AGRITECH "National Research Centre for Agricultural Technologies" `https://agritechcenter.it`, (Spoke 9) – CUP CN00000022, funded by "Piano Nazionale di Ripresa e Resilienza" (PNRR) - Next Generation EU.

Authors' contributions

Manlio Bacco: Conceptualization, Methodology, Original Draft, Review and Editing, Supervision, Funding Acquisition. Alexander Kocian: Original Draft, Review and Editing, Methodology, Investigation. Antonino Crivello: Original Draft, Review and Editing, Methodology, Investigation. Paolo Barsocchi: Funding Acquisition, Review and Editing. Stefano Chessa: Conceptualization, Original Draft, Review and Editing, Funding Acquisition.

References

[1] M. Franklin, A. Halevy, D. Maier, From Databases to Dataspaces: a new Abstraction for Information Management, ACM Sigmod Record 34 (4) (2005) 27–33.

[2] P. Ziegler, K. R. Dittrich, Data Integration—Problems, Approaches, and Perspectives, in: Conceptual modelling in information systems engineering, Springer, 2007, pp. 39–58.

[3] E. Curry, Dataspaces: Fundamentals, Principles, and Techniques, Real-time Linked Dataspaces: Enabling Data Ecosystems for Intelligent Systems (2020) 45–62.

[4] R. Cattell, Scalable SQL and NoSQL Data Stores, ACM Sigmod Record 39 (4) (2011) 12–27.

[5] P. Atzeni, F. Bugiotti, L. Cabibbo, R. Torlone, Data Modeling in the NoSQL World, Computer Standards & Interfaces 67 (2020) 103149.

[6] A. Halevy, M. Franklin, D. Maier, Principles of Dataspace Systems, in: Proceedings of the twenty-fifth ACM SIGMOD-SIGACT-SIGART symposium on Principles of database systems, 2006, pp. 1–9.

[7] M. Singh, S. Jain, A Survey on Dataspace, in: Advances in Network Security and Applications: 4th International Conference, CNSA 2011, Chennai, India, July 15-17, 2011 4, Springer, 2011, pp. 608–621.

[8] M. Singh, A Framework for Data Modeling and Querying Dataspace Systems, in: Seventh International Conference on Data Mining and Warehousing (ICDMW), 2013.

[9] A. D. Sarma, X. Dong, A. Y. Halevy, Data Modeling in Dataspace Support Platforms, Conceptual Modeling: Foundations and Applications: Essays in Honor of John Mylopoulos (2009) 122–138.

[10] C. Hedeler, K. Belhajjame, N. W. Paton, A. Campi, A. A. Fernandes, S. M. Embury, Chapter 7: Dataspaces, Search Computing: Challenges and Directions (2010) 114–134.

[11] Y. Wang, S. Song, L. Chen, A Survey on Accessing Dataspaces, ACM SIGMOD Record 45 (2) (2016) 33–44.

[12] A. Freitas, E. Curry, New Horizons for a Data-driven Economy: a Roadmap for Usage and Exploitation of Big Data in Europe, Springer International Publishing, 2016, Ch. Big Data Curation.

[13] A. Sexton, Dataspace: The Final Frontier, Springer, 2009.

[14] M. Franklin, A. Halevy, D. Maier, A First Tutorial on Dataspaces, Proceedings of the VLDB Endowment 1 (2) (2008) 1516–1517.

[15] S. Simmons, 1.09 - metadata and spatial data infrastructure, in: B. Huang (Ed.), Comprehensive Geographic Information Systems, Elsevier, Oxford, 2018, pp. 110–124. doi:https://doi.org/10.1016/B978-0-12-409548-9.09611-1.
URL https://www.sciencedirect.com/science/article/pii/B9780124095489096111

[16] A. Kotsev, M. Minghini, R. Tomas, V. Cetl, M. Lutz, From spatial data infrastructures to data spaces—a technological perspective on the evolution of european sdis, ISPRS International Journal of Geo-Information 9 (3) (2020) 176.

[17] C. Granell, S. Schade, G. Hobona, Spatial data infrastructures and linked data, Geospatial Web Services: Advances in Information Interoperability, IGI Global, P. Zhao (Editor) (2010).

[18] J. Crompvoets, National spatial data clearinghouses: worldwide development and impact, Wageningen University and Research, 2006.

[19] M. Di Leo, M. Bacco, A. Kona, M. Minghini, Self-Assessment Framework for Earth Observation Platforms from User Experience, The International Archives of the Photogrammetry, Remote Sensing and Spatial Information Sciences 48 (2024) 43–49.

[20] E. Curry, S. Scerri, T. Tuikka, Data Spaces: Design, Deployment and Future Directions, Springer Nature, 2022.

[21] B. Otto, M. ten Hompel, S. Wrobel, Designing Data Spaces: The Ecosystem Approach to Competitive Advantage, Springer Nature, 2022.

[22] A. V. Sambra, E. Mansour, S. Hawke, M. Zereba, N. Greco, A. Ghanem, D. Zagidulin, A. Aboulnaga, T. Berners-Lee, Solid: a Platform for Decentralized Social Applications based on Linked Data, MIT CSAIL & Qatar Computing Research Institute, Tech. Rep. (2016).

[23] W3C, Verifiable Credentials Data Model v1.1, Tech. rep. (2022).
URL https://www.w3.org/TR/vc-data-model

[24] Á. Alonso, A. Pozo, J. Cantera, F. de la Vega, J. Hierro, Industrial Data Space Architecture Implementation Using FIWARE, Sensors 18 (7) (2018) 2226.

[25] U. Ahle, J. J. Hierro, FIWARE for Data Spaces, Designing Data Spaces (2022) 395.

[26] EU, Simpl: Cloud-to-Edge Federations Empowering EU Data Spaces, Tech. rep. (2024).
URL https://digital-strategy.ec.europa.eu/en/policies/simpl

[27] M. J. Page, J. E. McKenzie, P. M. Bossuyt, I. Boutron, T. C. Hoffmann, C. D. Mulrow, L. Shamseer, J. M. Tetzlaff, E. A. Akl, S. E. Brennan, R. Chou, J. Glanville, J. M. Grimshaw, A. Hróbjartsson, M. M. Lalu, T. Li, E. W. Loder, E. 5o Wilson, S. McDonald, L. A. McGuinness, L. A. Stewart, J. Thomas, A. C. Tricco, V. A. Welch, P. Whiting, D. Moher, The PRISMA 2020 statement: an Updated Guideline for Reporting Systematic Reviews, Systematic Reviews 10 (1) (2021).

[28] T. Czvetkó, J. Abonyi, Data Sharing in Industry 4.0-AutomationML, B2MML and International Data Spaces-based Solutions, Journal of Industrial Information Integration 33 (2023) 100438.

[29] V. C. Gomes, G. R. Queiroz, K. R. Ferreira, An Overview of Platforms for Big Earth Observation Data Management and Analysis, Remote Sensing 12 (8) (2020) 1253.

[30] T. Usländer, A. Teuscher, Industrial Data Spaces, in: Designing Data Spaces, Springer, 2022.

[31] A. S. Ahmadian, J. Jürjens, D. Strüber, Extending Model-Based Privacy Analysis for the Industrial Data Space by Exploiting Privacy Level Agreements, in: Proceedings of the 33rd Annual ACM Symposium on Applied Computing, ACM, 2018.

[32] G. S. Brost, M. Huber, M. Weiß, M. Protsenko, J. Schütte, S. Wessel, An Ecosystem and IoT Device Architecture for Building Trust in the Industrial Data Space, in: Proceedings of the 4th ACM Workshop on Cyber-Physical System Security, ACM, 2018.

[33] S. Cuno, L. Bruns, N. Tcholtchev, P. Lämmel, I. Schieferdecker, Data Governance and Sovereignty in Urban Data Spaces Based on Standardized ICT Reference Architectures, Data 4 (1) (2019) 16.

[34] E. Curry, W. Derguech, S. Hasan, C. Kouroupetroglou, U. ul Hassan, A Real-time Linked Dataspace for the Internet of Things: Enabling Pay-As-You-Go Data Management in Smart Environments, Future Generation Computer Systems 90 (2019) 405–422.

[35] Y. Li, J. Li, P. Shi, X. Qin, Building an Open Cloud Virtual Dataspace Model for Materials Scientific Data, Intelligent Automation and Soft Computing (2019) 615–625.

[36] M. Niinimaki, P. Thanisch, Dataspace Management for Large Data Sets, in: Innovative Computing Trends and Applications, Springer International Publishing, 2019, pp. 13–21.

[37] B. Otto, M. Jarke, Designing a Multi-Sided Data Platform: Findings from the International Data Spaces Case, Electronic Markets 29 (4) (2019) 561–580.

[38] D. Sarabia-Jacome, I. Lacalle, C. E. Palau, M. Esteve, Enabling Industrial Data Space Architecture for Seaport Scenario, in: 2019 IEEE 5th World Forum on Internet of Things (WF-IoT), IEEE, 2019.

[39] F. Volz, L. Stojanovic, R. Lamberti, An Industrial Marketplace - the Smart Factory Web Approach and Integration of the International Data Space, in: 2019 IEEE 17th International Conference on Industrial Informatics (INDIN), IEEE, 2019.

[40] S. Wei, H. Zhihua, W. Zikai, Y. Zheng, D. Wei, A Method and Application for Constructing a Authentic Data Space, in: 2019 IEEE International Conference on Internet of Things and Intelligence System (IoTaIS), IEEE, 2019.

[41] F. Bruckner, J. Pampus, F. Howar, A Policy-Agnostic Programming Language for the International Data Spaces, in: Communications in Computer and Information Science, Springer International Publishing, 2021, pp. 172–194.

[42] D. Sarabia-Jacome, C. E. Palau, M. Esteve, F. Boronat, Seaport Data Space for Improving Logistic Maritime Operations, IEEE Access 8 (2020) 4372–4382.

[43] M. Nast, B. Rother, F. Golatowski, D. Timmermann, J. Leveling, C. Olms, C. Nissen, Work-in-Progress: Towards an International Data Spaces Connector for the Internet of Things, in: 2020 16th IEEE International Conference on Factory Communication Systems (WFCS), IEEE, 2020.

[44] C. Olms, C. Nissen, A. Schier, J. Leveling, R. Rademacher, M. Ten Hompel, Architektur einer adaptiven plattform für unternehmensübergreifende datenbasierte dienste mit dem international data spaces; [Architecture of an adaptive platform for cross-enterprise data-based services with the international data spaces], Logistics Journal 2020 (2020).

[45] J. Guo, Y. Cheng, D. Wang, F. Tao, S. Pickl, Industrial Dataspace for smart manufacturing: connotation, key technologies, and framework, International Journal of Production Research 61 (12) (2021) 3868–3883.

[46] J. Hernandez, L. McKenna, R. Brennan, TIKD: A Trusted Integrated Knowledge Dataspace For Sensitive Healthcare Data Sharing, in: 2021 IEEE 45th Annual Computers, Software, and Applications Conference (COMPSAC), IEEE, 2021.

[47] V. Janev, M. E. Vidal, K. Endris, D. Pujic, Managing Knowledge in Energy Data Spaces, in: Companion Proceedings of the Web Conference 2021, ACM, 2021.

[48] P. Li, K. Cheng, P. Jiang, Investigation on Quantitative Analysis of Carbon Footprint in Discrete Manufacturing by Using the Innovative Energy Dataspace Approach, Manufacturing Letters 27 (2021) 58–62.

[49] H. Qarawlus, M. Hellmeier, J. Pieperbeck, R. Quensel, S. Biehs, M. Peschke, Sovereign Data Exchange in Cloud-Connected IoT using International Data Spaces, in: 2021 IEEE Cloud Summit (Cloud Summit), IEEE, 2021.

[50] T. Werkmeister, Interaction Design Patterns for International Data Space-Based Ecosystems, in: Advances in Intelligent Systems and Computing, Springer International Publishing, 2021, pp. 315–322.

[51] T. Werkmeister, Development of User-Centred Interaction Design Patterns for the International Data Space, in: Design, User Experience, and

Usability: UX Research and Design, Springer International Publishing, 2021, pp. 144–155.

[52] H. Zeiner, R. Unterberger, Time-aware Data Spaces - A key Computing Unit in the Edge-to-Cloud Continuum, in: 2021 8th International Conference on Future Internet of Things and Cloud (FiCloud), IEEE, 2021.

[53] J. Doerr, R. Kalmar, B. Rauch, S. Stiene, Data Spaces in Agriculture - Status Quo and Perspectives, in: LAND.TECHNIK 2022, VDI Verlag, 2022, pp. 511–520.

[54] R. Kalmar, B. Rauch, J. Dörr, P. Liggesmeyer, Agricultural Data Space, Springer, 2022, Ch. Designing Data Spaces.

[55] D. R. Firdausy, P. de Alencar Silva, M. van Sinderen, M.-E. Iacob, A Data Connector Store for International Data Spaces, in: Cooperative Information Systems, Springer International Publishing, 2022, pp. 242–258.

[56] D. R. Firdausy, P. D. A. Silva, M. V. Sinderen, M.-E. Iacob, Towards a Reference Enterprise Architecture to Enforce Digital Sovereignty in International Data Spaces, in: 2022 IEEE 24th Conference on Business Informatics (CBI), IEEE, 2022.

[57] M. A. Inigo, J. Legaristi, F. Larrinaga, A. Perez, J. Cuenca, B. Kremer, E. Montejo, A. Porto, Towards Standardized Manufacturing as a Service through Asset Administration Shell and International Data Spaces Connectors, in: IECON 2022 – 48th Annual Conference of the IEEE Industrial Electronics Society, IEEE, 2022.

[58] J. Pampus, B.-F. Jahnke, R. Quensel, Evolving Data Space Technologies: Lessons Learned from an IDS Connector Reference Implementation, in: Lecture Notes in Computer Science, Springer Nature Switzerland, 2022, pp. 366–381.

[59] C. Stellmach, M. R. Muzoora, S. Thun, Digitalization of Health Data: Interoperability of the Proposed European Health Data Space, in: Studies in Health Technology and Informatics, IOS Press, 2022.

[60] A. J. Stoter, B. Rietveld, V. Jansen, H. J. Bastiaansen, Harmonization Profiles for Trusted Data Sharing Between Data Spaces: Striking the Balance between Functionality and Complexity, in: Proceedings of the Workshop of I-ESA'22, March 23–24, 2022, Valencia, Spain, Vol. 3214, 2022.

[61] V. Vassilev, D. Petrova-Antonova, T. Bali, S. Radu, V. Sowinski-Mydlarz, D. Mariyanayagam, E. Marinov, P. O. Hristov, S. Nakarmi, M. Rabka, Towards First Urban Data Space in Bulgaria, in: 2022 IEEE International Smart Cities Conference (ISC2), IEEE, 2022.

[62] R. Zhang, H. Yan, Z. Xiao, G. Zhou, F. Chang, S. Yang, X. Fei, X. Yang, Intelligent Manufacturing Core Enterprise-dominated Collaborative Dataspace Framework for New Energy Vehicle, in: 2022 International Conference on Data Analytics, Computing and Artificial Intelligence (ICDACAI), IEEE, 2022.

[63] R. Falcão, R. Matar, B. Rauch, F. Elberzhager, M. Koch, A Reference Architecture for Enabling Interoperability and Data Sovereignty in the Agricultural Data Space, Information 14 (3) (2023) 197.

[64] B. Farahani, A. K. Monsefi, Smart and Collaborative Industrial IoT: a Federated Learning and Data Space Approach, Digital Communications and Networks 9 (2) (2023) 436–447.

[65] S. Meckler, R. Dorsch, D. Henselmann, A. Harth, The Web and Linked Data as a Solid Foundation for Dataspaces, in: Companion Proceedings of the ACM Web Conference 2023, ACM, 2023.

[66] A. M. Schleimer, N. Jahnke, B. Otto, Architecture Design Options for Federated Data Spaces, in: Proceedings of the 56th Hawaii International Conference on System Sciences, Vol. 2023-January, 2023, p. 3643 – 3652.

[67] A. Turcan, Leveraging Data Spaces in Digital Twins for Smart Cities Deployment, in: Impact of Digital Twins in Smart Cities Development, IGI Global, 2023, pp. 80–102.

[68] J. Langford, A. Poikola, W. Janssen, V. Lähteenoja, M. Rikken, Understanding mydata operators, Tech. rep. (2020).
URL `https://www.mydata.org/wp-content/uploads/2022/03/Understanding-MyData-Operators-2022.pdf`

[69] A. Braud, G. Fromentoux, B. Radier, O. Le Grand, The Road to European Digital Sovereignty with Gaia-X and IDSA, IEEE network 35 (2) (2021) 4–5.

[70] A. Poikola, D. Wong, V. Lähteenoja, M. Turpeinen, STATE OF FINNISH DATA SPACES, Tech. rep., SITRA (2024).
URL https://media.sitra.fi/app/uploads/2024/04/state-of-finnish-data-spaces.pdf

[71] O. Boris, S. Sebastian, T. Andreas, B. Sebastian, et al., International Data Spaces (IDS) - Reference Architecture Model (RAM) 4.2.0, Tech. rep. (2023).
URL https://docs.internationaldataspaces.org/ids-knowledgebase/v/ids-ram-4/

[72] I. A. Machado, C. Costa, M. Y. Santos, Data Mesh: Concepts and Principles of a Paradigm Shift in Data Architectures, Procedia Computer Science 196 (2022) 263–271.

[73] W3C, Data Catalog Vocabulary, Tech. rep. (2024).
URL https://www.w3.org/TR/vocab-dcat-3

[74] B. Otto, A. Burmann, Europäische Dateninfrastrukturen: Ansätze und Werkzeuge zur Nutzung von Daten zum Wohl von Individuum und Gemeinschaft, Informatik Spektrum 44 (2021) 283–291.

[75] A. Gieß, M. Hupperz, T. Schoormann, F. Möller, What Does it Take to Connect? Unveiling Characteristics of Data space Connectors, in: Proceedings of the 57th Hawaii International Conference on System Sciences, 2024, pp. 4238–4247.

[76] International Data Spaces Association, Dataspace Protocol, Tech. rep. (2024).
URL https://docs.internationaldataspaces.org/ids-knowledgebase/v/dataspace-protocol

[77] M. Marina, F. Eimear, C. S. Bruno, P. S. Monica, S. Serena, V. Michele, Mapping the Landscape of Data Intermediaries, Tech. rep., European Commission, Joint Research Centre (Seville) (2023).

[78] I. D. S. Association, IDSA connector, https://international-data-spaces-association.github.io/DataspaceConnector/GettingStarted, [Online; accessed 23-March-2024] (2024).

[79] sovity GmbH, Connecting to data space, https://sovity.de/en/connect-to-data-space-en/, [Online; accessed 23-March-2024] (2024).

[80] IDSA, Certification, https://internationaldataspaces.org/offers/certification/, [Online; accessed 23-March-2024] (2024).

[81] E. Foundation, Eclipse Dataspace Components (EDC), https://projects.eclipse.org/projects/technology.edc, [Online; accessed 23-March-2024] (2024).

[82] IDSA, Data Connector Report, Tech. rep. (2024).
URL https://internationaldataspaces.org/wp-content/uploads/dlm_uploads/IDSA-Data-Connector-Report-88-No-12-February-2024-4.pdf

[83] J.-P. Dittrich, M. A. V. Salles, iDM: a Unified and Versatile Data Model for Personal Dataspace Management, in: Proceedings of the 32nd international conference on Very large data bases, Citeseer, 2006, pp. 367–378.

[84] M. A. V. Salles, J.-P. Dittrich, S. K. Karakashian, O. R. Girard, L. Blunschi, iTrails: Pay-as-you-go Information Integration in Dataspaces, in: VLDB, Vol. 7, 2007, pp. 663–674.

[85] E. Curry, W. Derguech, S. Hasan, C. Kouroupetroglou, U. ul Hassan, A Real-Rime Linked Dataspace for the Internet of Things: Enabling "Pay-as-You-Go" Data Management in Smart Environments, Future Generation Computer Systems 90 (2019) 405–422.

[86] B. Otto, J. Jürjens, J. Schon, S. Auer, N. Menz, S. Wenzel, J. Cirullies, Industrial Data Space. Digital Souvereignity Over Data (2016).

[87] DSSC, , Tech. rep. (2024).
URL https://dssc.eu/space/News/blog/381878275/Introducing+Blueprint+1.0%3A+the+evolution+of+Data+Spaces

[88] S. Chessa, G. M. Dimitri, M. Gori, A. Kocian, WoA: An Infrastructural, Web-Based Approach to Digital Agriculture, in: P. Novais, V. Julián Inglada, M. J. Hornos, I. Satoh, D. Carneiro, J. Carneiro, R. S. Alonso (Eds.), Proc. 14th International Symposium on Ambient Intelligence (ISAmI), Springer Nature Switzerland, Cham, 2023, pp. 113–122.
